# Intrasubtype Reassortments Cause Adaptive Amino Acid Replacements in H3N2 Influenza Genes

**DOI:** 10.1371/journal.pgen.1004037

**Published:** 2014-01-09

**Authors:** Alexey D. Neverov, Ksenia V. Lezhnina, Alexey S. Kondrashov, Georgii A. Bazykin

**Affiliations:** 1 Federal Budget Institution of Science “Central Research Institute for Epidemiology”, Moscow, Russia; 2 Department of Bioengineering and Bioinformatics, M.V. Lomonosov Moscow State University, Moscow, Russia; 3 Life Sciences Institute and Department of Ecology and Evolutionary Biology, University of Michigan, Ann Arbor, Michigan, United States of America; 4 Institute for Information Transmission Problems of the Russian Academy of Sciences (Kharkevich Institute), Moscow, Russia; Fred Hutchinson Cancer Research Center, United States of America

## Abstract

Reassortments and point mutations are two major contributors to diversity of Influenza A virus; however, the link between these two processes is unclear. It has been suggested that reassortments provoke a temporary increase in the rate of amino acid changes as the viral proteins adapt to new genetic environment, but this phenomenon has not been studied systematically. Here, we use a phylogenetic approach to infer the reassortment events between the 8 segments of influenza A H3N2 virus since its emergence in humans in 1968. We then study the amino acid replacements that occurred in genes encoded in each segment subsequent to reassortments. In five out of eight genes (NA, M1, HA, PB1 and NS1), the reassortment events led to a transient increase in the rate of amino acid replacements on the descendant phylogenetic branches. In NA and HA, the replacements following reassortments were enriched with parallel and/or reversing replacements; in contrast, the replacements at sites responsible for differences between antigenic clusters (in HA) and at sites under positive selection (in NA) were underrepresented among them. Post-reassortment adaptive walks contribute to adaptive evolution in Influenza A: in NA, an average reassortment event causes at least 2.1 amino acid replacements in a reassorted gene, with, on average, 0.43 amino acid replacements per evolving post-reassortment lineage; and at least ∼9% of all amino acid replacements are provoked by reassortments.

## Author Summary

Influenza A is a rapidly evolving virus with genome composed of eight distinct RNA molecules called segments. This genetic structure allows formation of new combinations of segments when a cell is coinfected by multiple viral strains, in a process called reassortment. While “antigenic drift” – the process of continuous accumulation of point mutations that change the antigenic properties of the viral proteins – is mainly responsible for the seasonal flu, the heaviest pandemics were caused by spread of novel reassortant strains and the associated radical “antigenic shift”. However, the association between these two types of processes has not been studied systematically. Here, we use the extensive available complete-genome sequencing data for Influenza A H3N2 subtype to infer the evolutionary timings of within-subtype reassortment events, and study the patterns of point amino acid-changing replacements that followed reassortments. We find that reassortments were often rapidly followed by replacements, which possibly compensated for the loss of fitness associated with the reassortment or explored newly accessible fitness peaks. These findings may be relevant for prediction of future pandemic strains of Influenza A.

## Introduction

The genome of influenza A virus consists of 8 segments, each represented by an RNA molecule. Coinfection of a cell by viruses of different genotypes occasionally leads to reassortments, i. e. formation of genotypes containing molecules from different sources. Most of the major Influenza A pandemics during the last century were caused by reassortant strains [1]. Indeed, reassortments, especially those creating novel combinations of hemagglutinin (HA) and neuraminidase (NA) genes, may lead to radical changes in antigenic properties and give rise to viral types that escape the herd immunity. Still, after a reassortment event, the viral segments find themselves in a novel genetic environment, which may lead to disruption of coadaptations that previously existed between them and reduce viral fitness [2], [3].

Thus, it is likely that only a small proportion of reassortment events lead to creation of novel, successful viral genotypes. Reassortments between different Influenza A subtypes gave rise to the major pandemics of the 1957, 1968, 2009, and possibly 1918 [1], [4]. Reassortments between strains belonging to a single subtype likely occur much more frequently than inter-subtype reassortments; however, they leave a less pronounced phylogenetic signal, and are therefore harder to study [5].

In theory, reassortments can be detected through the incongruencies between phylogenies of different segments of a viral genome. Indeed, after a reassortment, the segments obtained from the same viral isolate will occupy conflicting phylogenetic positions, due to the differences in their evolutionary histories. In practice, however, detecting reassortments is difficult. The influenza sequence databases are subject to ascertainment biases, with recent sequences being oversampled, and some countries sampled better than others. Reassortment events are prone to be missed when one or both parental strains are not sampled properly, or when they are closely related. Conversely, spurious reassortments may be inferred due to differences in phylogenies caused by phylogenetic noise. Multiple reassortments nested within a single clade compound the difficulties.

In most of the early studies, reassortments were inferred via manual detection of incongruencies between phylogenies of different viral segments [6]–[9]. However, this approach is impractical for systematic analyses of large datasets of influenza genomes with complex reassortment histories. Recently, several methods for automatic detection of reassortments have been proposed. These methods can be broadly categorized into two groups. The distance methods [10], [11] measure, for each viral segment, the degree of similarity between all pairs of viral genomes, and infer reassortments from the differences between the distance matrices obtained from different segments. The phylogenetic methods [4], [12]–[14] make explicit use of the evolutionary histories of individual segments, comparing their phylogenies and detecting incompatibilities between them. In general, phylogenetic methods are more robust than the distance methods [12], [14], particularly in detecting reassortments that became fixed or reached high frequencies within the population.

Comparisons of relative frequencies of different progeny coming from coinfecting strains in experiments [3],[15]–[20] as well as phylogenetic analyses of circulating reassortant strains [10], [21] demonstrate that reassortments between different segments are not equiprobable. Such differences likely arise, in part, from variance in the extent of epistatic interactions between pairs of genes. For example, a recent analysis of experimental data suggests that polymerase genes (PB1 and PA) tend to be inherited together, and that reassortment preferences for HA depend on the subtypes of the parental strains, while NA and matrix protein (MP) have no preferences in their reassortments with other segments [19]. Analysis of distributions of coalescent times for different segments suggests, however, that reassortments between HA and NA are particularly frequent [22].

Besides radical shifts in antigenic properties, reassortments are associated with a reduction in genetic diversity of circulating strains, indicative of positive selection favoring the spread of the reassortant strain [22]. Several observations also suggest that, subsequent to the “antigenic shift”, reassortants tend to undergo increased “antigenic drift”, i.e., elevated rate of amino acid replacements, perhaps due to follow-up coadaptation of genes that find themselves in new genetic environments [5], [23]. For example, a reassortment associated with host change has led to short-term positive selection in NS gene of swine Influenza A [24]. Still, the phenomenon of coevolution of viral genes subsequent to reassortments has not yet been studied systematically. Here, we purport to close this gap.

## Results

### Identification of reassortments

To study the within-subtype reassortments in the influenza H3N2 virus, we first used GiRaF [12] to automatically infer the reassortant taxa in the dataset of 1376 complete influenza H3N2 genotypes. GiRaF compares large pools of Monte Carlo-sampled phylogenetic trees constructed for each viral segment separately, inferring the topological incongruencies between them. Assuming that an incongruence between phylogenies of two segments, observed in a high fraction of comparisons, reflects an ancestral reassortment event, GiRaF then predicts the subsets of taxa that are descendant to such reassortments. The number of reassortments predicted by GiRaF in which a particular segment was involved was similar for all segments except M1 and NS1 (Table 1); in M1 and NS1, fewer reassortments were detected, probably because their shorter sequences and/or higher conservation (Table 1) lead to a weaker phylogenetic signal. We saw no preferences for particular pairs of segments to be reassorted (Table S1).

**Table 1:** Characteristics of RCBs and amino acid replacements.

	PB2	PB1	PA	HA	NP	NA	M1	NS1
Mean dN/dS	0.07	0.07	0.08	0.27	0.07	0.25	0.05	0.35
Sequence length, nucleotides	2271	1986	2142	1692	1485	1377	687	471
RCBs								
Internal	19	20	21	16	19	20	12	7
With descendant substitutions	6	11	10	10	9	11	3	5
Without descendant substitutions	6	4	3	1	4	3	3	0
Pre-terminal*	7	5	8	5	6	5	6	2
Terminal	15	16	16	13	15	16	6	7
Internal branches with substitutions								
All	125	117	139	271	91	257	27	89
Descendant to RCBs	104	99	105	223	68	216	14	68
Substitutions on internal branches								
All	154	146	173	460	118	411	29	107
Descendant to RCBs	127	123	127	336	83	320	14	77
Distance between post-RCB substitutions and most recent RCB, dS units								
Mean								
expected	0.050	**0.043**	0.027	0.036	0.029	**0.030**	0.024	0.038
observed	0.047	**0.034**	0.026	0.035	0.032	**0.026**	0.021	0.038
Median								
expected	0.028	**0.019**	0.023	0.029	0.022	**0.020**	0.024	0.040
observed	0.028	**0.017**	0.020	0.026	0.028	**0.016**	0.023	0.049
Wilcoxon one-tailed p-value	0.211	**0.034**	0.149	0.106	0.952	**1.8×10^−5^**	0.196	0.065

The mean distances between the amino acid replacements and most recent RCBs that were significantly (p<0.05) lower than expected are in boldface.

Pre-terminal branches are those immediately ancestral to the terminal branches.

**Figure 1:**
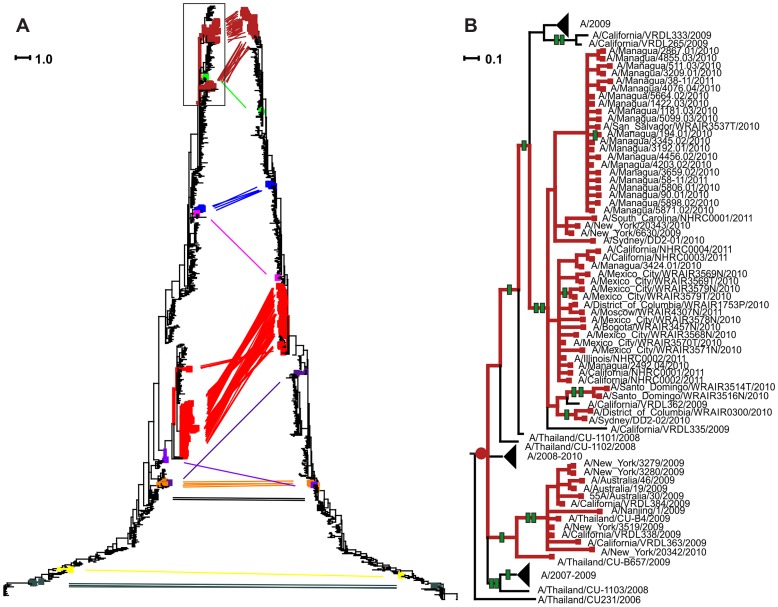
Reassortment events inferred from comparison of phylogenies of HA and NA segments of Influenza A H3N2 virus, and subsequent accumulation of amino acid replacements. (A) The tanglegram [77] of the NA (left) and HA (right) genes for the same set of strains. Topologies of the phylogenies of the two genes are incongruent; such incongruence can be resolved by assuming reassortments. The clades corresponding to inferred reassortments are shown in color, with the lines of the corresponding color connecting the inferred reassortants for the two genes. (B) Zoom-in of the clade in rectangle in A. Red dot, inferred RCB (node id 62); green hatches, amino acid replacements on internal branches following the RCB. Some clades are collapsed into black triangles for clarity, and substitutions on such clades are not shown.

We then mapped the reassortments inferred by GiRaF onto the reconstructed phylogenies of each of the segments involved. We assumed that each reassortment event had happened on the phylogenetic branch leading to the last common ancestor of the reassortant taxa (reassortment-carrying branch, RCB). This way of mapping reassortments was self-evident for monophyletic sets of taxa. However, when the histories of reassortment events are complex (which is the case for Influenza A; [7], [9]), the subsets of genotypes resulting from a single reassortment predicted by GiRaF are not necessarily monophyletic [12]. Indeed, we found that in our dataset, many of the inferred sets of reassortant taxa were not monophyletic (Figure 1). To test the robustness of our conclusions, we therefore also tried alternative ways of mapping reassortment events, which also supported our key findings (see below).

### Elevated rate of amino acid replacements after reassortments

We asked whether reassortments affect subsequent accumulation of amino acid-changing replacements. We hypothesized that subsequently to RCBs, the rate of accumulation of amino acid replacements will be temporarily elevated as the reassorted sets of genes coadapt to each other.

To address this, we inferred, for the genes coded by each of the 8 segments, the phylogenetic positions of all amino acid replacements, and studied those replacements that were descendant to at least one RCB. When a reassortment and a replacement occurred on the same phylogenetic branch, it was impossible to deduce which came first. To avoid this ambiguity, we considered a replacement to be descendant to a particular reassortment if it occurred on a phylogenetic branch descendant to an RCB, but not on the RCB itself. (Including the substitutions on the RCBs as descendants to reassortments gave similar results; see below.) Between 33% and 50% (depending on the segment) of the RCBs were terminal branches, and thus their effect on subsequent replacements could not be studied. 14% to 33% more of the RCBs were pre-terminal branches. Amino acid replacements on the terminal branches, especially in RNA viruses, tend to be more deleterious, and may follow evolutionary patterns distinct from the replacements elsewhere on the phylogeny [25]–[30]; thus, we chose to exclude such replacements from our analyses. Therefore, the RCBs on pre-terminal branches could also have no effect on replacements in our dataset. The remaining 33% to 42% of RCBs could be followed by amino acid replacements, and in fact, most of the replacements occurred in reassortant clades. In NA, for example, 320 out of 411 (78%) observed non-terminal amino acid replacements were descendant to at least one RCB (Table 1).

To assess the effect of reassortments on subsequent accumulation of amino acid replacements, we measured the phylogenetic distance between each replacement and its most recent ancestral RCB. We then compared these distances to those expected if the phylogenetic positions of post-reassortment replacements were random in respect to reassortments. This approach is conservative, in that it ignores any possible long-term effect of reassortments spanning phylogenetic distances comparable with the height of the phylogenetic tree.

In two of the genes, NA and PB1, the mean distance was significantly lower than expected (Table 1), indicating that RCBs were followed by a transient increase in the rate of amino acid replacements. Since the inferred number of RCBs for each gene is moderate (e.g., 20 for NA, only 14 of which could have descendant replacements), individual RCBs could have a disproportionate effect on the distribution of distances. For the NA segment, we asked whether the observed accelerated evolution after reassortments is due to some single reassortment event. To this end, we repeated the analysis 14 times, each time excluding one of the RCBs, and comparing the observed and expected distances between the amino acid replacements and the remaining RCBs. In all 14 comparisons, the results remained significant, indicating that the acceleration of replacements is a phenomenon to which multiple reassortments contribute (Table 2).

**Table 2:** Reassortments involving NA.

		Distance between post-RCB substitutions and their most recent ancestral RCB, excluding the current RCB, dS units					Reassortment-provoked substitutions very soon after reassortments^a^			
Node id	No. descendant substitutions	Mean, observed	Mean, expected	Median, observed	Median, expected	Wilcoxon one-tailed p-value	Total	Phylogenetically independent^b^	Number of lineages	Length of adaptive walk^c^
1090^e^	32	0.0347	0.0355	0.0208	0.0224	0.02616	0	0	18	0.00
62^e^	77	0.0318	0.0353	0.0256	0.0305	0.00045	26	12	27	0.96
49	78	0.0454	0.0519	0.0191	0.0223	0.00011	2	1	4	0.50
1092^d^ ^e^	19	0.0265	0.0302	0.0190	0.0208	0.00005	0	0	6	0.00
57^d^ ^e^	49	0.0391	0.0446	0.0233	0.0324	0.00004	0	0	1	0.00
257	5	0.0261	0.0298	0.0176	0.0207	0.00004	0	0	1	0.00
35	9	0.0260	0.0299	0.0160	0.0192	0.00002	0	0	3	0.00
38	42	0.0300	0.0338	0.0168	0.0208	0.00002	0	0	1	0.00
986	4	0.0260	0.0298	0.0160	0.0203	0.00002	0	0	1	0.00
1446^d^	1	0.0261	0.0299	0.0160	0.0207	0.00002	0	0	1	0.00
952^d^	4	0.0260	0.0298	0.0160	0.0203	0.00002	2	2	2	1.00
602	0	0.0260	0.0298	0.0160	0.0203	0.00001	0	0	1	0.00
941	0	0.0260	0.0299	0.0160	0.0203	0.00001	0	0	1	0.00
1433	0	0.0260	0.0299	0.0160	0.0207	0.00001	0	0	2	0.00
All	320	0.0260	0.0297	0.0160	0.0203	0.00002	30	15	69	0.43

The 14 non-terminal, non-pre-terminal RCBs are shown. The table shows the observed and expected distances between the amino acid replacements and the preceding reassortment, and the Wilcoxon p-value for the difference between the observed and expected values. The RCBs are ordered by the Wilcoxon p-value.

^a^ Substitutions at phylogenetic distances up to 0.003 ds units after the reassortment.

^b^ Phylogenetically independent substitutions are such that none of them are descendant to any of the remaining ones.

^c^ Number of substitutions accumulated per lineage.

^d^ RCBs also described in [7].

^e^ RCBs confirmed by sampling times.

To further test its robustness, we repeated the analyses using alternative methods of mapping reassortments onto the tree (Tables S2, S3, S4, S5, S6, S7). These methods differ in the strength of the required statistical evidence for inference of reassortments, and in the ways non-monophyletic sets of reassortant taxa are treated (see below). For NA, acceleration of evolution after reassortments was significant in 5 out of 6 analyses; in the sixth analysis, it was marginally significant (p = 0.09). For PB1, acceleration was significant in 3 out of 6 analyses. Moreover, in a number of analyses, acceleration was also observed in several other genes for which no significant result is observed in Table 1: M1 in 4 tests, and HA and NS1 in 3 tests each. Thus, 5 out of 8 genes show evidence for acceleration at least in half of the tests. Still, the results for NA are the most robust, and this is the gene for which the evidence for reassortments-caused-acceleration of evolution is the strongest. When the substitutions on the RCB itself were also counted as descendant to the reassortment, the results were similar (Tables S8, S9, S10, S11, S12, S13).

**Figure 2:**
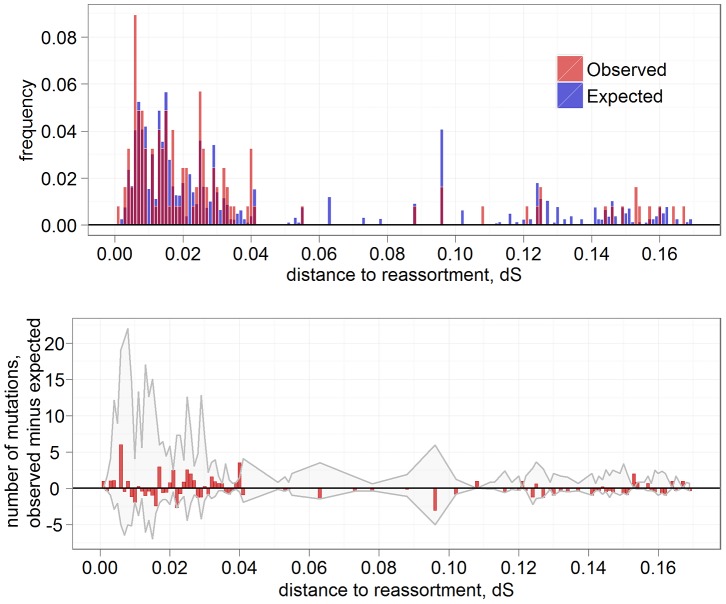
Excess of amino acid replacements after RCBs in PB1. Top: frequencies of amino acid replacements observed at various phylogenetic distances from the most recent ancestral reassortment; pink: observed; gray: expected; red: overlayed observed and expected. Bottom: the excess of amino acid replacements, compared to the expectation. Gray shaded area indicates the 90% CI for the expected value. The expected distribution was obtained from 10,000 reshuffling trials.

**Figure 3:**
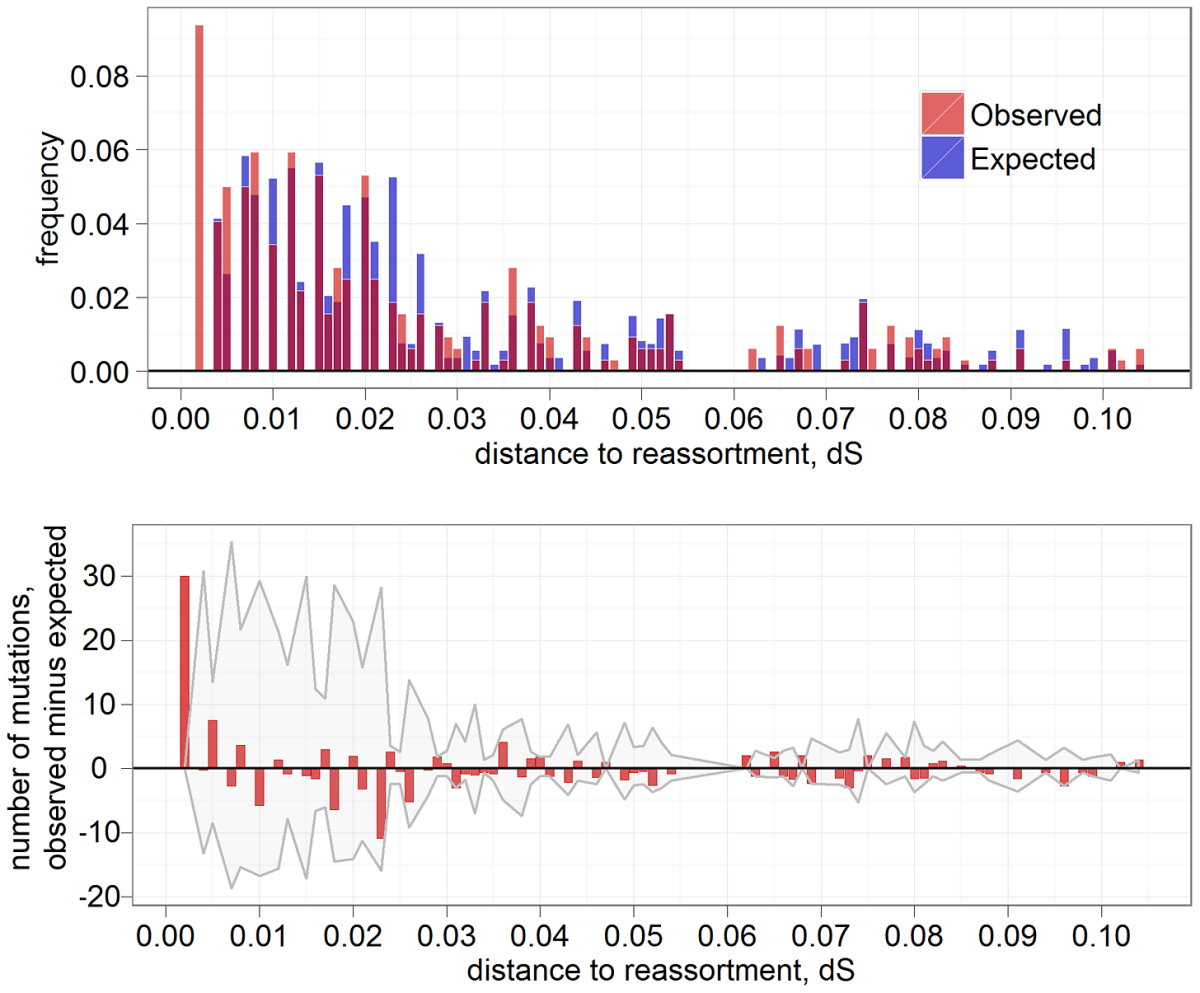
Excess of amino acid replacements after RCBs in NA. Notations as in Figure 2.

In PB1, the increase in the rate of amino acid replacements after the RCB is rather long-lived: it spans a phylogenetic distance of ∼0.04 ds units, although the excess is not significant for most individual distance bins (Figure 2). In contrast, in NA, this increase is very brief, with most of the excess replacements observed on the very short phylogenetic branches that immediately follow the RCB (Figure 3). In particular, we observe 30 such replacements at phylogenetic distances up to ∼0.003 ds units (which is just above the time it takes the NA gene to obtain a single synonymous replacement, and is therefore the highest phylogenetic resolution we can achieve; leftmost bin in Figure 3). Because virtually no such replacements would be expected to occur in such a short period of time if they had been independent of reassortments, all these excess 30 replacements are reassortment-provoked. Therefore, at least ∼9% (30/320) of all amino acid replacements in NA were caused by reassortments; since some of the later replacements descendant to the RCBs could also be reassortment-provoked, the actual number is probably higher. Moreover, the fact that these replacements occur so fast implies that most of them are facilitated by positive selection, and thus comprise a post-reassortment adaptive walk [31], [32].

What is the length of such adaptive walks, i.e., the characteristic number of amino acid replacements provoked by an individual reassortment? The 30 “fast” replacements were descendant to 3 individual RCBs (Table 2); 11 more RCBs were not followed by replacements so soon, although they could be followed by reassortment-associated replacements later. Therefore, an average reassortment provoked ∼2.1 (30/14) amino acid replacements in its descendant clade. However, these replacements could occur in multiple independent lineages, so that the number of post-reassortment replacements per lineage was lower. Indeed, by the time the phylogenetic distance of 0.003 ds units after an RCB was reached, the descendant subtree had often multifurcated, so that these 3 RCBs gave rise to a total of 33 individual descendant lineages; 36 more lineages originated from the remaining 11 RCBs. In 2 out of the 3 RCBs that provoked replacements, different post-RCB lineages accumulated replacements independently (Table 2). As a result, over the evolutionary time of 0.003 ds units, an average post-RCB lineage attained 0.43 (30/69) reassortment-provoked replacements (Table 2). This is the lower boundary for the length of the “adaptive walk” per lineage associated with a reassortment event, as it excludes any effect of reassortments over longer timescales.

### Distances to ancestral RCBs for different classes of amino acid replacements

We asked whether the post-RCB amino acid replacements are enriched in particular classes of mutations, compared to the rest of the replacements. In this analysis, we considered the NA gene, because in it, the effect of post-reassortment adaptive walk is the most robust; and also the HA gene, because it is the other primary determinant of antigenic properties, is known to evolve under continuous positive selection, and is highly epidemiologically relevant.

Several categories of mutations had biased phylogenetic distances from the RCBs, compared with the complementary sets (Table 3). Firstly, in NA, the replacements at amino acid sites experiencing positive selection tended to be farther from RCBs, while no such difference was observed for HA. Secondly, the replacements at sites that distinguish the antigenic clusters [33] of HA tended to occur farther from RCBs. Thirdly, parallel replacements had a strong tendency to occur soon after the RCBs both in NA and HA. Fourthly, reversions in NA, but not in HA, occurred soon after the RCBs. Fifthly, the sites previously shown to be involved in intragenic epistatic interactions [34] as “leading” occurred father from RCBs both in NA and in HA, while the “trailing” sites occurred soon after the RCBs. Neither the NA nor the HA genes showed any bias for replacements at the epitopic sites. As was the case with the previous analysis, most of these results were also supported by alternative methods of inferring RCBs (Tables S14, S15, S16, S17, S18, S19); the exception were reversions in NA, which gave discordant results in different tests.

**Table 3:** Phylogenetic distances from reassortments for different classes of amino acid replacements.

			Distance between post-RCB nonsynonymous substitutions and most recent RCB, dS units				
Gene	Subset	No. substitutions	Mean, subset	Mean, complement	Median, subset	Median, complement	Wilcoxon one-tailed p-value
NA	*Positively selected*	*44*	*0.0328*	*0.0249*	*0.0241*	*0.0160*	*0.003*
	Epitopes	88	0.0257	0.0261	0.0160	0.0176	0.772
	**Parallel**	**188**	**0.0229**	**0.0303**	**0.0145**	**0.0199**	**0.004**
	**Reversions**	**53**	**0.0213**	**0.0269**	**0.0096**	**0.0191**	**0.007**
	*Epistatic leading*	*95*	*0.0325*	*0.0233*	*0.0205*	*0.0145*	*0.001*
	**Epistatic trailing**	**143**	**0.0226**	**0.0287**	**0.0112**	**0.0195**	**0.002**
HA	Positively selected	91	0.0317	0.0366	0.0238	0.0291	0.165
	Epitopes	215	0.0342	0.0372	0.0251	0.0277	0.273
	*Antigenic*	*102*	*0.0395*	*0.0335*	*0.0329*	*0.0251*	*0.017*
	**Parallel**	**159**	**0.0314**	**0.0388**	**0.0224**	**0.0330**	**0.003**
	Revertions	60	0.0315	0.0361	0.0221	0.0269	0.218
	*Epistatic leading*	*151*	*0.0385*	*0.0327*	*0.0329*	*0.0238*	*0.040*
	Epistatic trailing	169	0.0357	0.0349	0.0251	0.0274	0.898

The phylogenetic distances to the most recent ancestral RCB are compared between a given class of amino acid replacements and all remaining replacements. Classes that are significantly (p<0.05) closer to the most recent RCB are in boldface, and classes that are farther from RCB, in italic.

## Discussion

Our study provides the first systematic analysis of association between reassortments and amino acid-level changes in influenza A. We show that a reassortment involving a particular segment provokes a transient increase in the rate of amino acid replacements at the gene encoded on this segment; these replacements tend to occur at sites that do not normally experience positive selection, and often involve parallel replacements.

One way to estimate the effect of RCBs on subsequent accumulation of amino acid replacements would be to directly compare the replacement rates on branches that had descended from reassortments and those that had not. However, this is not feasible in Influenza A, because in most segments, the majority of branches (50–85%) descend from at least one RCB (Table 1); the remaining branches tended to be deep-lying, and thus likely have biased patterns of replacements [29], [30]. Instead, we searched for a transient increase in the rate of amino acid changes on the branches descendant to the internal RCBs. This analysis could miss some of the very rapid reassortment-provoked changes that had happened on the same branches as the reassortments themselves.

We compared the phylogenetic distances between the amino acid replacements and the preceding RCBs to the null distribution expected under the assumption that the amino acid replacements were distributed over post-RCB, non-terminal branches randomly, with probability of a replacement to fall onto a particular branch proportional to the branch length. In NA and PB1 genes, the replacements occur sooner after the RCBs than in the null model. When alternative strategies of mapping reassortments were used, three other genes – M1, HA and NS1 – also show the same pattern in at least half of the tests. Since it is hard to validate the reassortment-mapping algorithms, the results of each individual analysis for each individual gene should be taken with some caution. Together, however, they provide strong support for the reassortment-caused accumulation of changes, especially in the NA gene. The post-RCB excess of amino acid replacements was not exclusively dependent on a single particular RCB (Table 2); thus, it seems to be a universal phenomenon.

Non-uniformity of the substitution rate has been described previously for Influenza, and has been attributed to episodic action of positive selection [35], to simultaneous fixation of multiple interacting advantageous mutations [36] or to frequent selective sweeps under clonal interference. There is no obvious mechanistic association between reassortments and either of these factors. If reassortments themselves are adaptive, they can spread through population rapidly by means of positive selection [22]. Strong positive selection favoring an allelic variant may also drive to fixation neutral and even mildly deleterious point mutations linked with it [37]; this phenomenon is prevalent in Influenza A which evolves, on the within-segment level, under nearly complete linkage [38], and where clonal dynamics is largely determined by linkage with beneficial alleles [38], [39]. This phenomenon could cause an excess of replacements on the same branches as the reassortment events, which is, however, not observed in our data (data not shown). There is no way hitchhiking can cause accumulation of replacements on the branches descendant to RCBs.

Therefore, the only feasible explanation for the post-reassortment increase of the rate of amino acid replacements seems to be that they constitute an adaptive walk [31], [32], i.e., a burst of positively selected adaptive changes provoked by a shift in the fitness landscape. In NA, we observe a radical increase in the rate of amino acid replacements immediately after reassortments. This excess spans only a short period of time: it is mostly over by 0.003 ds units, i.e., by the time a single synonymous replacement occurs somewhere in the gene (which takes less than a year; [40]).

Thus, at least NA, and very likely other genes, experience transient positive selection after reassortment events. The excess of replacements at phylogenetic distances up to 0.003 ds (i.e., up to the time a single neutral replacement is expected to be accumulated at the gene) in NA suggest that a total of ∼30 amino acid replacements in the entire phylogeny, or ∼0.43 replacements per lineage, were facilitated by preceding RCBs. Therefore, reassortments are responsible for at least ∼9% of all amino acid replacements in this gene. In fact, this may be an underestimate for at least two reasons. First, some of the reassortments were likely to have been undetected. Second, a fraction of the adaptive walks could have spanned longer phylogenetic distances than this threshold (Figures 2, 3).

What is the cause of the post-reassortment accumulation of positively selected replacements? Influenza A is a model system for studying positive selection, with most of the selective pressure exerted by the host immune system. Positive selection is most pronounced in the genes coding for the surface glycoproteins (NA and HA), and within these genes, at the epitopic sites which are most involved in the immune response. Conceivably, the immunity-driven positive selection could increase after a reassortment. We observe, however, that the post-RCB adaptive walk is mostly manifested at sites other than those under constant positive selection, or responsible for antigenic properties, and is not affected by the epitopic vs. non-epitopic location of the site (Table 3). This suggests that the post-reassortment adaptive walks are not driven by the pressure to evade the host immune system.

Rather, these replacements are probably associated with epistatic interactions between genes [41]. In general, reassortments and host shifts lead to changes in the patterns of both synonymous and nonsynonymous substitutions, probably due to joint effects of changes in the mutation and selection patterns [29], [42]–[44]. After a reassortment event, a gene finds itself in a novel genetic environment which may, through epistatic interactions, exert novel selective pressures on its amino acid sites, facilitating further amino acid changes. An adaptive walk could compensate for the loss of fitness associated with the preceding reassortment [2]; however, as the reassortments themselves tend to be adaptive [22], it seems more likely that these replacements could exploit novel fitness peaks that have become newly accessible after the reassortments.

The evidence for the post-reassortment adaptive walk is the most robust for the NA gene. Sequence evolution of influenza NA does not always lead to changes in antigenic properties [45], and may be caused by other forces instead. Indeed, antibody-driven affinity-changing mutations in HA can be compensated by substitutions changing the activity of NA [46], [47]; this indicates that the choice of the optimal NA genotype is dependent at least on HA, and possibly on other genes as well. Furthermore, reassortments often involve a currently circulating strain and an older strain [48], and a reassortment between HA and NA frequently involves an up-to-date variant of HA and an older variant of NA, as suggested by less discordance between sampling time and phylogenetic position of HA sequences than of NA sequences ([7] and our data). Therefore, while the immune escape is the primary factor of evolution of HA, much of the NA evolution may be epistatic and, in particular, compensatory.

Parallel replacements are overrepresented after reassortments (Table 3). Overall, the rate of parallelism in Influenza A evolution is high [49], [50], probably due to similar selective pressures exerted on different strains. The high parallelism observed in this study suggests that the replacements involved in a post-reassortment adaptive walk may also be adaptive in other contexts.

Epistatic interactions both within [51]–[54] and between segments [55] are wide-spread in Influenza A. One evolutionary manifestation of this phenomenon is positive epistasis between replacements: a replacement can facilitate subsequent replacements at different sites of the same protein [34]. The sites involved in such epistatic interactions can be classified as “leading” or “trailing”, depending on whether replacements in them tend to come as first or second in epistatic pairs; for example, replacements at leading sites can introduce radical changes to protein structure, while replacements at trailing sites may compensate those changes [34]. We find that, while the replacements at leading sites are remote from reassortments, the replacements at trailing sites occur sooner after reassortments than expected. Therefore, the sites experiencing post-reassortment replacements are the same sites that also react to the change of the protein structure due to replacements elsewhere in the protein. This suggests that the class of sites denoted as “trailing” in [34], and involved in post-adaptive walk in this study, may be responsible for adaptation to novel genetic environment that stems from changes in the same gene as well as in other genes.

Association between reassortments and the rate of subsequent accumulation of amino acid mutations may be important for predicting future pandemic strains. For example, the avian H5N1 influenza is among the most likely candidates for the agent of a future pandemia [56]–[58]. Naturally occurring strains of A/H5N1 are not transmittable between mammals; however, to become transmittable, they require just five additional mutations [59] or a reassortment with just four additional mutations [60]. Two of these mutations are already frequent among the A/H5N1 viruses [61]. If a reassortment commonly leads to accelerated accumulation of amino acid replacements, gaining the remaining mutations and evolving a natural mammalian-transmittable H5N1 strain may take less time than predicted by simple models [61].

## Methods

### Sequences and alignments

We downloaded all complete human H3N2 influenza A genotype sequences (N = 2205) available on 27.10.2011 from the flu database [62]. Nucleotide sequences for each segment were aligned using muscle [63], [64]. Genotypes containing truncated sequences, multiple unidentified nucleotides, or indels were discarded. We used CD-HIT [65] to cluster genotypes that had identical sequences of NA segments, and retained one random sequence from each cluster, thus retaining 1379 genotypes for further analysis. For segments encoding PB1, M1 and NS1 that contain overlapping ORFs, we excluded the overlapping regions, and analyzed the longest remaining ORF. All alignments are available at http://makarich.fbb.msu.ru/flu_walks/.

### Phylogenetic analysis

For each segment, we Bayes-sampled the 1,000 phylogenetic trees using MrBayes MPI version [66]–[68] with the following settings: GTR+I+G model, 22 million iterations, sampling each 22,000th iteration. Three isolates: A/Ontario/RV123/2005, A/Ontario/1252/2007 and A/Indiana/08/2011 were excluded from analyses, because we found the branch leading to the clade formed by them to be, for several segments of non-human origin (NP, M, NS, PB2 and PA), too long for a meaningful estimation of evolutionary parameters; these isolates are SOIV triple reassortants (see also [69]). Each phylogenetic tree was rooted by the isolate A/Albany/18/1968. These 1,000 trees were used to infer the reassortment events (see below).

For each segment, the consensus tree of the 1,000 MrBayes-sampled trees was used as input for HyPhy [70] to estimate the evolutionary parameters and to restore the ancestral sequences. The branch-specific dS values were estimated using the local MG94xHKY85 [71] model. The ancestral sequences were reconstructed using the GTR+I+G global nucleotide model. As an alternative approach, we also repeated our analyses using maximum likelihood trees constructed with PhyML [72] instead on consensus Bayesian trees, and obtained similar results.

For subsequent analyses, we rescaled the lengths of all branches in the consensus trees in the units of dS; this allowed us to study the distribution of nonsynonymous replacements independently of branch lengths. To obtain the gene-specific dN/dS values, we used global MG94xHKY85 model in HyPhy.

### Inference of reassortments

For phylogenetic mapping of reassortments, we used a two-step procedure. First, we computationally predicted the subsets of taxa that occupied incompatible positions in phylogenies of different segments using GIRAF software [12] running on a cluster node with 512 Gb of RAM. GiRaF automatically predicted the subsets of taxa originating from each ancestral reassortment event on the basis of the MrBayes sampled trees for all eight segments.

Importantly, not all segments were necessarily involved in each inferred reassortment; although in reality each reassortment splits all segments into two subsets (the retained and the acquired segments), for some of the segments, the phylogenetic signal was often too weak to allow GiRaF to ascribe them to one of the two mutually reassorting sets of segments [12]. For such segments, no reassortment event could then be inferred; as a result, although the same sets of taxa were considered for each segment, the number of reassortment events per segment varied (Table 1), and the number of segments involved in each reassortment (on either side) was usually under 8, with some of the segments “abstaining”. We used two approaches to quality filtering of the predicted reassortments. In the first approach (“joint reassortments”, recommended in [12] and used in the main text), we acquired the subsets of taxa involved in reassortments from the “catalog file” produced by GiRaF. This file includes only those reassortments that involved inconsistencies between at least 3 pairs of segments; therefore, each reassortment involved between 4 and 8 segments. In the second approach (“high-confidence reassortments”), we used the reassortment subsets involving any number of pairs of segments (i.e., between 2 and 8 segments), but required the GiRaF-predicted confidence level for the reassortments of 1.0.

Second, we inferred, on the basis of these lists of reassortant taxa, the phylogenetic positions of the RCBs. In theory, each reassortment event should give rise to a monophyletic set of taxa; the last common ancestral branch to this clade is then the RCB. In reality, however, many of the predicted subsets of taxa were not monophyletic. This occurs because, under complex histories of sequential reassortments, GIRAF can either split the taxa descendant to a common reassortment event into multiple sets, or join the taxa descendent from multiple reassortment events into a single set [12]. In such cases, the inference of RCBs is ambiguous.

We used three different approaches to infer the phylogenetic position of the RCBs. While these approaches produced identical results for monophyletic sets of reassortants, they differed in the way they treated non-monophyletic sets. For each set of reassortants, we inferred as RCB(s) (i) the (single) branch leading to the most recent common ancestor of all reassortants (“one-point inference”, used in the main text); (ii) the set of branches leading to the most recent common ancestors of each clade involving only reassortants (“two-point inference”); or (iii) the union of (i) and (ii) (“three-point inference”). Arguably, each approach has its merits. Under (i), the number of inferred RCBs is minimal (and equal to the number of sets of reassortant taxa), and so this approach is most parsimonious; conversely, under (ii) and (iii), a single subset of reassortant taxa could give rise to multiple RCBs on the same phylogenetic tree. Under (ii), only reassortant taxa are descendants to RCBs; finally, (iii) may be best for inference of sequences of nested reassortments such that a later reassortment affects a subset of lineages that were also involved in an earlier reassortment, and GIRAF underpredicts the set of reassortant taxa for the earlier reassortment.

All GiRaF output files and the phylogenetic trees in Nexus FigTree (http://tree.bio.ed.ac.uk/software/figtree/) format with reassortments mapped onto them are available at http://makarich.fbb.msu.ru/flu_walks/.

### Validation of reassortments

For NA, we used two approaches for validation of the observed reassortments. First, we compared our RCBs with the reassortments inferred previously on the basis of manual analysis of a much smaller dataset [7]. Out of the 6 reassortments reported in [7], 5 mapped precisely to 4 of our RCBs (accounting for the strains missing in [7], and including one pre-terminal RCB; two of the reassortments mapped to the same RCB). The remaining reassortment mapped to a branch adjacent to an RCB (Table S20). The reassortments from [7] are included for comparison with our RCBs in the phylogenetic trees available at http://makarich.fbb.msu.ru/flu_walks/.

Second, we analyzed the inconsistencies in the dates of sampling of strains. In general, the sampling dates of Influenza strains are highly correlated with their distance from the root on the “cactus-shaped” Influenza phylogeny [33], consistently with the major role of continuous positive selection on immunity avoidance shaping it [73]. However, recombination may lead to disruptions of this order, because a strain nested deep in the phylogeny may have a recent sampling date if it has reassorted with another recent strain.

We inferred the inconsistencies in the sampling dates as follows (the approach is similar to that used in [48]). We split all our strains (including those identical by NA sequence) into subsets depending on their most recent RCB, and further into smaller subsets based on the year of sampling. We then built a consensus sequence for each of these subsets, excluding those that carried fewer than 10 sequences, and used these consensuses to construct a new ML phylogenetic tree with branches scaled in ds units as described above. We then rotated the branches of the tree to order them by the sampling year. Some of the branches could not be thus ordered; i.e., had sampling dates inconsistent with their phylogenetic position. Specifically, seven of the branches, descendant to four inferred reassortments, had sampling dates later than some of the branches found to the right of them on the tree (Figure S1), supporting their origin from reassortment events. The reassortments that corresponded to these branches were among the top-ranking in our analysis (Table 2). The method of inference of reassortments based on inconsistencies in sampling dates of a single segment is orthogonal to our main approach based on inconsistencies between phylogenies of different segments; therefore, it provides an independent validation for the reassortments that we detect.

### Phylogenetic distribution of nonsynonymous replacements

Using the reconstructed ancestral sequences, we inferred, for each segment, the phylogenetic positions of all nonsynonymous replacements. We then measured the distance between each nonsynonymous replacement and its ancestral reassortment. If multiple RCBs were ancestral to a given replacement, we considered the most recent one. In measurements of phylogenetic distances, we assumed that reassortments occurred at the middles of the RCBs, and that each nonsynonymous replacement occurred at the middle of the corresponding phylogenetic branch. We used two approaches for dealing with replacements on the RCBs themselves: they were considered to be either ancestral to this reassortment, and thus excluded from the list of its descendants (Tables 1–3, S2, 3, S4, S5, S6, S7, S14, S15, S16, S17, S18, S19); or descendant to this reassortment, with the distance between the reassortment and the replacement equal to zero (Tables S8, S9, S10, S11, S12, S13). The distances were measured in dS units.

To obtain the expected phylogenetic distribution of the replacements, we, in 10,000 Monte Carlo trials, redistributed the replacements among the tree branches. The probability of a replacement to fall onto a given branch was taken to be proportional to its dS value. We excluded from reshuffling the branches that were not descendant to at least one RCB, the terminal branches, and (for Tables 1–3, S2, S3, S4, S5, S6, S7, S14, S15, S16, S17, S18, S19) the RCBs themselves; since replacements on those branches are not included in corresponding analyses, the number of analyzed replacements was thus conserved.

For the analysis of the length of adaptive walk in NA, we considered a replacement reassortment-provoked if it occurred within 0.003 ds units after reassortment. We traced the number of phylogenetic lineages descendant to each RCB at this time point, and calculated the per-lineage length of the adaptive walk by dividing the number of replacements by the number of lineages. Post-RCB replacements were considered phylogenetically independent if none of them were descendant to any of the remaining ones; the number of such replacements equaled the number of lineages carrying replacements at distance of 0.003 ds.

### Subsets of sites and classes of mutations in HA and NA

Positively selected sites were inferred by IFEL [70] and MEME [74] methods from the HyPhy package; a site was considered positively selected if it was predicted by either of these methods. Epitopic sites were taken from [34], and sites distinguishing the HA antigenic clusters, from [33]. Replacements from a particular ancestral amino acid to a particular descendant one that occurred at more than one lineage on the phylogeny were categorized as parallel, and replacements that reverted to a once-ancestral state, reversing.

All manipulations with phylogenetic trees were done using the Perl Bio::Phylo package [75]. The statistical analyses were performed with R [76].

## Supporting Information

Figure S1 Validation of reassortments based on sampling dates for NA gene. The ML tree of consensus sequences was constructed as described in the Methods. The branches that had sampling dates inconsistent with their phylogenetic position are in green.
(PDF)

Table S1 Involvement of individual segments in the inferred reassortment events. For each of the 28 unordered pairs of 8 segments, the number of times these two segments were reassorted together (‘cis’) or split by reassortment (‘trans’) is provided, together with the Fisher's exact test p-value for deviation from independence. The pairs of segments are ordered by p-values. None of the p-values are significant after Bonferroni correction (p>0.1).
(DOC)

Table S2 Characteristics of RCBs and amino acid replacements. Joint reassortments, one-point inference (same as Table 1).
(XLS)

Table S3 Characteristics of RCBs and amino acid replacements. Joint reassortments, two-point inference.
(XLS)

Table S4 Characteristics of RCBs and amino acid replacements. Joint reassortments, three-point inference.
(XLS)

Table S5 Characteristics of RCBs and amino acid replacements. High confidence reassortments, one-point inference.
(XLS)

Table S6 Characteristics of RCBs and amino acid replacements. High confidence reassortments, two-point inference.
(XLS)

Table S7 Characteristics of RCBs and amino acid replacements. High confidence reassortments, three-point inference.
(XLS)

Table S8 Characteristics of RCBs and amino acid replacements. Joint reassortments, one-point inference; substitutions on RCBs included as descendant to reassortment.
(XLS)

Table S9 Characteristics of RCBs and amino acid replacements. Joint reassortments, two-point inference; substitutions on RCBs included as descendant to reassortment.
(XLS)

Table S10 Characteristics of RCBs and amino acid replacements. Joint reassortments, three-point inference; substitutions on RCBs included as descendant to reassortment.
(XLS)

Table S11 Characteristics of RCBs and amino acid replacements. High confidence reassortments, one-point inference; substitutions on RCBs included as descendant to reassortment.
(XLS)

Table S12 Characteristics of RCBs and amino acid replacements. High confidence reassortments, two-point inference; substitutions on RCBs included as descendant to reassortment.
(XLS)

Table S13 Characteristics of RCBs and amino acid replacements. High confidence reassortments, three-point inference; substitutions on RCBs included as descendant to reassortment.
(XLS)

Table S14 Phylogenetic distances from reassortments for different classes of amino acid replacements. Joint reassortments, one-point inference (same as Table 3). Classes that are significantly (p<0.05) closer or farther from the most recent RCB are in boldface, with red color indicating closer to RCB, and blue, farther from RCB.
(XLS)

Table S15 Phylogenetic distances from reassortments for different classes of amino acid replacements. Joint reassortments, two-point inference. Classes that are significantly (p<0.05) closer or farther from the most recent RCB are in boldface, with red color indicating closer to RCB, and blue, farther from RCB.
(XLS)

Table S16 Phylogenetic distances from reassortments for different classes of amino acid replacements. Joint reassortments, three-point inference. Classes that are significantly (p<0.05) closer or farther from the most recent RCB are in boldface, with red color indicating closer to RCB, and blue, farther from RCB.
(XLS)

Table S17 Phylogenetic distances from reassortments for different classes of amino acid replacements. High confidence reassortments, one-point inference. Classes that are significantly (p<0.05) closer or farther from the most recent RCB are in boldface, with red color indicating closer to RCB, and blue, farther from RCB.
(XLS)

Table S18 Phylogenetic distances from reassortments for different classes of amino acid replacements. High confidence reassortments, two-point inference. Classes that are significantly (p<0.05) closer or farther from the most recent RCB are in boldface, with red color indicating closer to RCB, and blue, farther from RCB.
(XLS)

Table S19 Phylogenetic distances from reassortments for different classes of amino acid replacements. High confidence reassortments, three-point inference. Classes that are significantly (p<0.05) closer or farther from the most recent RCB are in boldface, with red color indicating closer to RCB, and blue, farther from RCB.
(XLS)

Table S20 Comparison of reassortments inferred in [7] with our RCBs.
(XLS)
