# Supplementary figures and images for: Intrasubtype Reassortments Cause Adaptive Amino Acid Replacements in H3N2 Influenza Genes
Source: PLoS Genet. 2014 Jan 9;10(1):e1004037. doi: 10.1371/journal.pgen.1004037 (PMC3886890; doi:10.1371/journal.pgen.1004037)

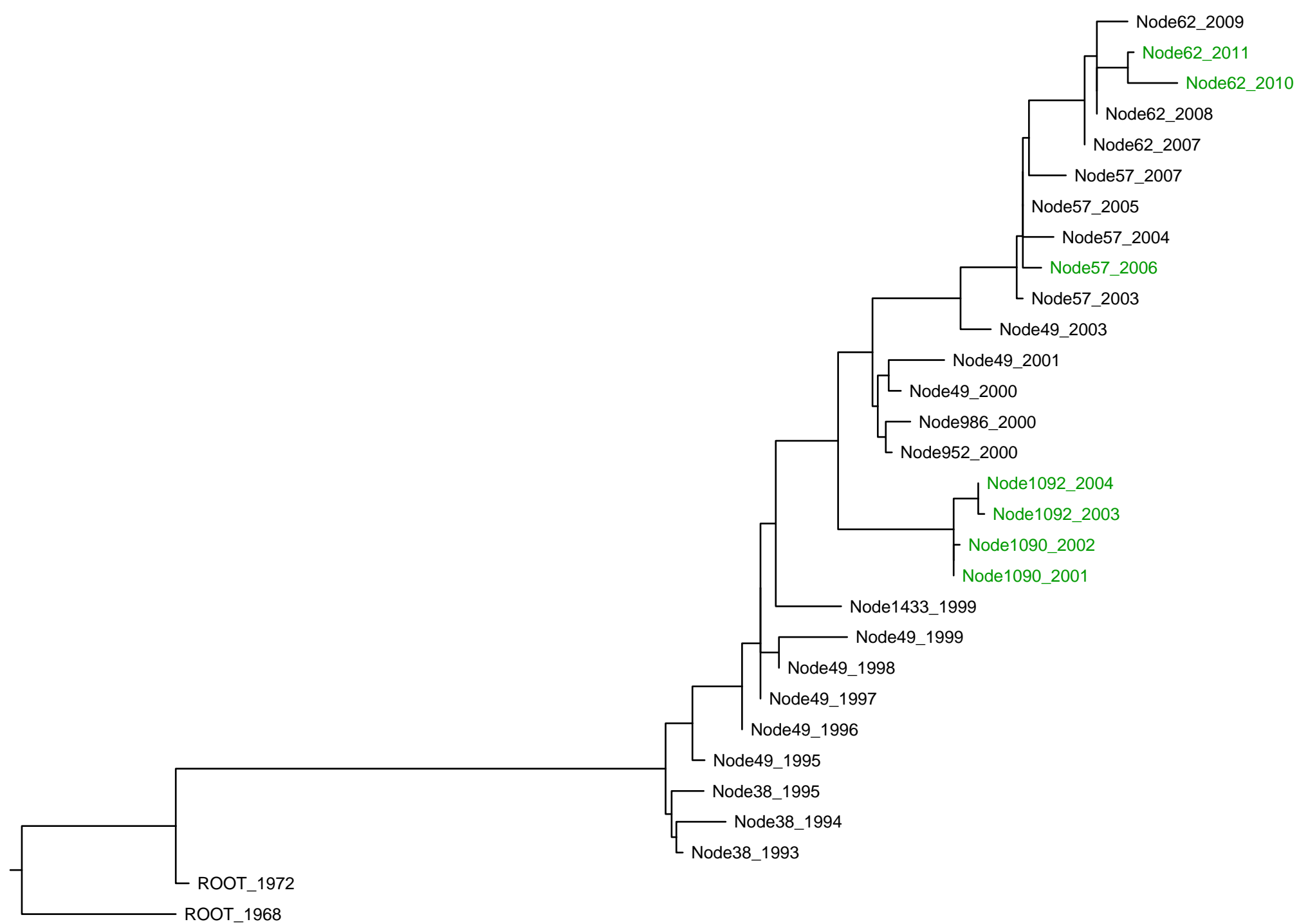

0.04

Supplement: Figure S1 — Validation of reassortments based on sampling dates for NA gene. The ML tree of consensus sequences was constructed as described in the Methods. The branches that had sampling dates inconsistent with their phylogenetic position are in green. (PDF) [file pgen.1004037.s001.pdf]
