# Supplementary material for: Intrasubtype Reassortments Cause Adaptive Amino Acid Replacements in H3N2 Influenza Genes
Source: PLoS Genet. 2014 Jan 9;10(1):e1004037. doi: 10.1371/journal.pgen.1004037 (PMC3886890; doi:10.1371/journal.pgen.1004037)
Supplement: Table S1 — Involvement of individual segments in the inferred reassortment events. For each of the 28 unordered pairs of 8 segments, the number of times these two segments were reassorted together (‘cis’) or split by reassortment (‘trans’) is provided, together with the Fisher's exact test p-value for deviation from independence. The pairs of segments are ordered by p-values. None of the p-values are significant after Bonferroni correction (p>0.1). (DOC) [file pgen.1004037.s002.doc]

**Table S1. Involvement of individual segments in the inferred reassortment events.**

| **Segment 1** | **Segment 2** | **Cis** | **Trans** | **p-value** |
| --- | --- | --- | --- | --- |
| M1 | NP | 13 | 3 | 0.004 |
| NP | NS1 | 10 | 2 | 0.006 |
| M1 | NS1 | 5 | 1 | 0.023 |
| NP | PB2 | 18 | 8 | 0.031 |
| NS1 | PA | 9 | 3 | 0.033 |
| NP | PA | 19 | 10 | 0.064 |
| NA | PA | 18 | 11 | 0.129 |
| NP | PB1 | 16 | 10 | 0.151 |
| M1 | PA | 11 | 7 | 0.188 |
| HA | PB1 | 15 | 10 | 0.195 |
| M1 | PB2 | 8 | 6 | 0.297 |
| NA | PB1 | 18 | 14 | 0.313 |
| M1 | NA | 9 | 7 | 0.322 |
| NS1 | PB2 | 6 | 5 | 0.360 |
| HA | NP | 12 | 10 | 0.381 |
| M1 | PB1 | 8 | 7 | 0.407 |
| HA | NA | 13 | 12 | 0.486 |
| NA | NS1 | 7 | 7 | 0.503 |
| NA | NP | 12 | 12 | 0.562 |
| PA | PB1 | 14 | 14 | 0.580 |
| HA | PA | 10 | 13 | 0.783 |
| HA | PB2 | 10 | 13 | 0.783 |
| M1 | HA | 4 | 8 | 0.871 |
| NA | PB2 | 12 | 17 | 0.878 |
| PB2 | PB1 | 11 | 17 | 0.914 |
| NS1 | PB1 | 5 | 11 | 0.941 |
| PA | PB2 | 10 | 18 | 0.960 |
| HA | NS1 | 2 | 8 | 0.968 |
|  | **Total** | **305** | **264** |  |

For each of the 28 unordered pairs of 8 segments, the number of times these two segments were reassorted together (‘cis’) or split by reassortment (‘trans’) is provided, together with the Fisher’s exact test p-value for deviation from independence. The pairs of segments are ordered by p-values. None of the p-values are significant after Bonferroni correction (p>0.1).
